# Isolated middle colic artery injury after blunt abdominal trauma

**DOI:** 10.31744/einstein_journal/2018AI4384

**Published:** 2018-10-30

**Authors:** Eduardo Kaiser Ururahy Nunes Fonseca, Fernando Ide Yamauchi, Milton Steinman, Thais Caldara Mussi, Adriano Tachibana, Ronaldo Hueb Baroni

**Affiliations:** 1 Hospital Israelita Albert Einstein, São Paulo, SP, Brazil.

A 34-year old male patient, victim of motorcycle accident at about 56 miles/hour, was brought into emergency care with a cervical collar. He was hemodynamically stable, with no respiratory or neurological impairment, complaining of diffuse moderate intensity abdominal pain during the physical examination. During secondary evaluation, abrasions were identified on the abdominal wall in the right flank area.

**Figure 1:**
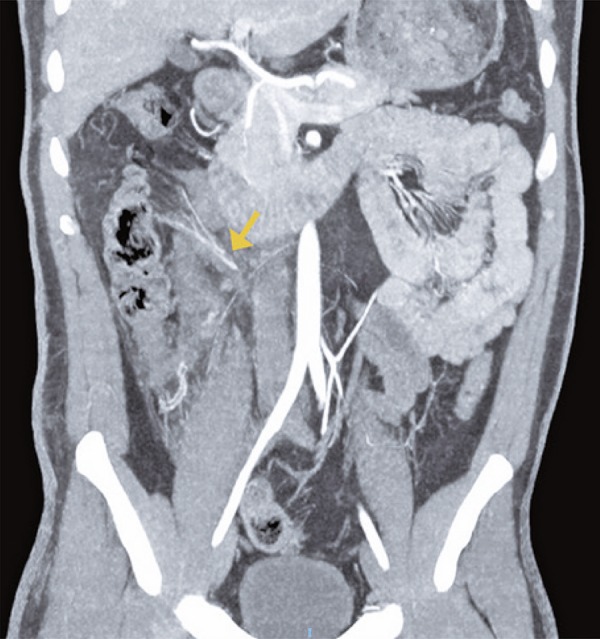
Coronal reconstruction of computed tomography in angiographic phase in maximum intensity projection, showing parietal irregularity and hypoenhancement in the middle and distal segments of a right colic vessel branch (arrow)

**Figure 2:**
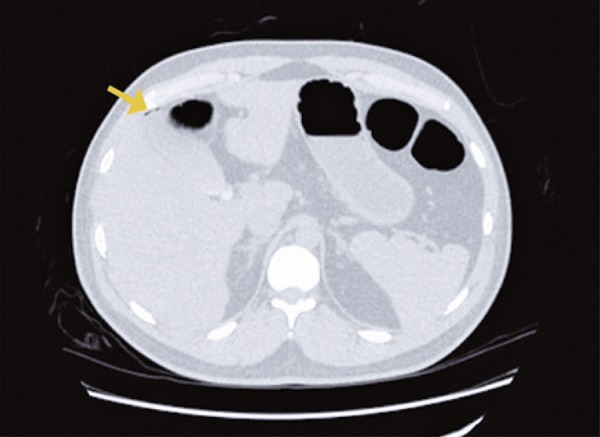
Axial computed tomography showing minimal pneumoperitoneum (arrow)

**Figure 3:**
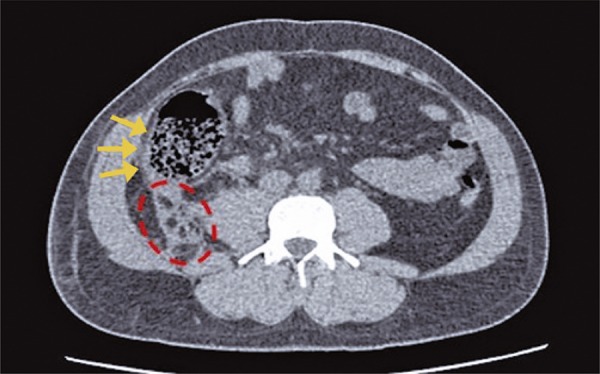
Axial computed tomography showing hemoperitoneum (circle) and right colon parietal thickening (arrows)

Due to high energy trauma, he underwent a whole body computed tomography (CT) that showed an hematoma in the mesenteric fat planes of the abdomen, and also filling defects in branches of the middle colic artery ( Figure 1 ). The presumptive preoperative diagnosis was vascular mesenteric lesion due to straining. The patient was stabilized, and initially conservative treatment was chosen. During the serial physical examination, persistence of abdominal pain was noted, with no signs of peritonitis. A new CT was performed 20 hours after the first, which revealed dilation and parietal thickening of the right colon, associated with a minimal pneumoperitoneum ( Figures 2 and 3 ).

**Figure 4:**
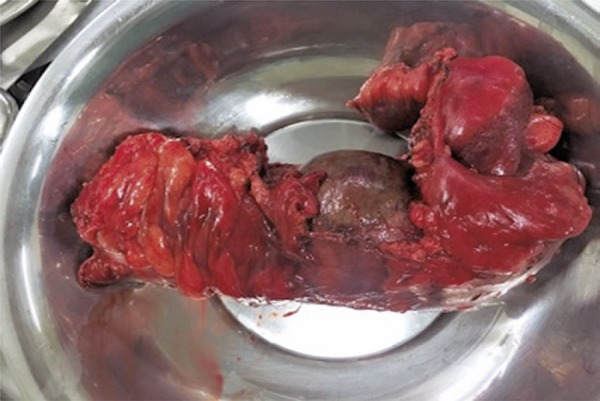
Surgical specimen photograph of right hemicolectomy

The patient was taken to the operating room and laparoscopy confirmed tomographic findings of mesenteric hematoma and acute ischemic colitis resulting from traumatic mesentery lesion. Right hemicolectomy, was performed ( Figure 4 ).

Blunt abdominal trauma is a frequently found event in the context of emergencies, in which three fourths of cases result from automobile accidents.^(^ ^1^ ^)^ In most cases, there is solid viscera involvement, especially spleen and liver; and isolated lesions of the mesentery are rare.^(^ ^1^ ^,^ ^2^ ^)^

It is believed that these cases result from deceleration forces, which lead to vascular strain, with rupture or mesenteric thrombosis and subsequent intestinal ischemia.^(^ ^3^ ^)^

The symptoms are generally non-specific, and not always reliable within the context of trauma. The diagnosis depends on whole body CT. The main findings can be divided into intestinal alterations resulting from ischemia, such as parietal thickening, decreased parietal enhancement, pneumatosis, and/or direct observation of the mesenteric injury, including filling defects, leakage of the contrast medium, mesenteric hematomas, and densification of adipose layers. Other findings also include intra-abdominal fluid collections and pneumoperitoneum.^(^ ^4^ ^-^ ^7^ ^)^

This case illustrates a rare vascular lesion in trauma. Although clinical signs are nonspecific and imaging findings subtle, is highly lethal if not promptly identified and treated. Whole body computed tomography with intravenous contrast is the gold standard in the evaluation of lesions related to trauma, including visceral and vascular lesions.
